# Clinical Outcomes Based on Measurable Residual Disease Status in Patients with Core-Binding Factor Acute Myeloid Leukemia: A Systematic Review and Meta-Analysis

**DOI:** 10.3390/jpm10040250

**Published:** 2020-11-26

**Authors:** Wannaphorn Rotchanapanya, Peter Hokland, Pattaraporn Tunsing, Weerapat Owattanapanich

**Affiliations:** 1 Division of Hematology, Department of Medicine, Chiangrai Prachanukroh Hospital, Chiang Rai 57000, Thailand; rot.wannaphorn@gmail.com; 2 Division of Hematology, Department of Clinical Medicine, Aarhus University Hospital, 8200 Aarhus N, Denmark; phokland@clin.au.dk; 3 Division of Hematology, Department of Medicine, Faculty of Medicine Siriraj Hospital, Mahidol University, Bangkok 10700, Thailand; sakorn.tun@mahidol.ac.th

**Keywords:** clinical outcomes, minimal residual disease status, core-binding factor acute myeloid leukemia, systematic review and meta-analysis

## Abstract

Measurable residual disease (MRD) response during acute myeloid leukemia (AML) treatment is a gold standard for determining treatment strategy, especially in core-binding factor (CBL) AML. The aim of this study was to critically review the literature on MRD status in the CBF-AML to determine the overall impact of MRD status on clinical outcomes. Published studies in the MEDLINE and EMBASE databases from their inception up to 1 June 2019 were searched. The primary end-point was either overall survival (OS) or recurrence-free survival (RFS) between MRD negative and MRD positive CBF-AML patients. The secondary variable was cumulative incidence of relapse (CIR) between groups. Of the 736 articles, 13 relevant studies were included in this meta-analysis. The MRD negative group displayed more favorable recurrence-free survival (RFS) than those with MRD positivity, with a pooled odds ratio (OR) of 4.5. Moreover, OS was also superior in the MRD negative group, with a pooled OR of 7.88. Corroborating this, the CIR was statistically significantly lower in the MRD negative group, with a pooled OR of 0.06. The most common cutoff MRD level was 1 × 10^−3^. These results suggest that MRD assessment should be a routine investigation in clinical practice in this AML subset.

## 1. Introduction

Intensive induction chemotherapy with subsequent consolidation chemotherapy and/or hematopoietic stem cell transplantation (HSCT) has been the standard therapy in acute myeloid leukemia (AML) for decades [1]. AML patients with chromosomal translocations that result in chimeric protein formations are diagnosed as having core-binding factor (CBF) AML, and the chromosomal translocations are located at t (8;21) and t (16;16) [2]. These fusion genes account for 15% of all adult AML, and they are associated with more favorable prognosis compared to other AML subtypes [3]. Patients with this favorable cytogenetic profile may continue with an additional 2–4 cycles of consolidation chemotherapy, whereas patients with other risks should be candidates for HSCT. Approximately 40–60% of CBF-AML patients have long-term survival from consolidation chemotherapy with high-dose cytarabine after complete remission (CR) [4]. Monitoring of molecular response, which is known as measurable residual disease (MRD), is a recently developed method for identifying the presence of leukemic cells to white blood cells at ratios ranging from 1:10^3^ to 1:10^6^ compared to 1:20 in morphological CR evaluation [5].

Monitoring of MRD response in AML is currently recommended in clinical trials and clinical practice [6]. This molecular response can be evaluated after induction, during consolidation therapy, or after completion of consolidation treatment to assess for disease relapse [7]. AML genetic marker can be measured by reverse transcription quantitative polymerase chain reaction (RT-qPCR) or multicolor flow cytometry (MFC) [8].

MRD assessment has been routinely employed in large multicenter trials for clinical decision-making to determine response outcomes in acute lymphoblastic leukemia (ALL) [9]. In AML MRD treatment, however, it has yet to be considered as standard disease status assessment in routine clinical practice [10]. An explanation for the latter can be that the MRD cutoff level for positivity as well as the time point at which MRD response is evaluated among AML patients has varied among previously published studies [11]. Thus, there is no recommendation guideline for MRD investigation in CBF-AML. Accordingly, the aim of this study was to review and pool the data on MRD status in CBF-AML and to determine the impact of MRD status on clinical outcomes.

## 2. Materials and Methods

### 2.1. Data Sources and Searches

This study aimed to determine the impact of MRD status on clinical outcomes in CBF-AML by searching published studies in the MEDLINE and EMBASE databases from their inception to 1 June 2019. The search terms included acute myeloid leukemia, core-binding factor, favorable, minimal residual disease, and minimal measurable disease, as listed in Supplementary Data 1. The references of the some identified articles and review articles were manually evaluated to search for additional eligible articles. The search and data collection processes were performed by two investigators (W.R. and W.O.). This study was conducted according to the Preferred Reporting Items for Systematic Reviews and Meta-Analyses (PRISMA) statement [12] (Supplementary Data 2).

### 2.2. Selection Criteria and Data Extraction

In order to be eligible for inclusion in the meta-analysis, studies needed to be randomized controlled studies or cohort studies, and both prospective and retrospective studies were reviewed. Study subjects needed to be CBF-AML patients who were compared between their MRD positive status and MRD negative status, and the MRD evaluation had to be by PCR method. The primary outcome of this systematic review and meta-analysis study was overall survival (OS) or recurrence-free survival (RFS) between MRD negative and MRD positive CBF-AML patients. The secondary outcome was cumulative incidence of relapse (CIR) between MRD groups. Two aforementioned investigators independently reviewed the eligibility of each study. If there was any disagreement, a consensus decision was reached between the two investigators.

### 2.3. Definition of Outcomes

OS was defined as duration from AML diagnosis to the time of death from any cause. RFS was defined as the time from CR to the time of AML recurrence or death. CIR was defined as the number of recurrent AML divided by the number of patients at risk during a defined period of time.

### 2.4. Quality Assessment

The eligible non-randomized articles were evaluated for their quality using the Newcastle–Ottawa Scale [13].

### 2.5. Statistical Analysis

We pooled the effect estimates and 95% confidence intervals (Cis) from each included article using the Mantel–Haenszel method [14]. Cochran’s Q test and the I^2^ statistic were used to evaluate statistical heterogeneity among the included studies. The categories of heterogeneity included insignificant heterogeneity (I^2^ value of 0–25%), low heterogeneity (I^2^ value: 26–50%), moderate heterogeneity (I^2^ value: 51–75%), and high heterogeneity (I^2^ value: 76–100%) [15]. We used a random effects model rather than a fixed effects model due to the high likelihood of between-study heterogeneity. Funnel plots to detect publication bias were not used due to the relatively small number of studies in each outcome. A *p*-value of less than 0.05 was defined as being statistically significant. Review Manager 5.3 software from the Cochrane Collaboration (London, UK) was applied for all statistical analyses.

## 3. Results

**Figure 1:**
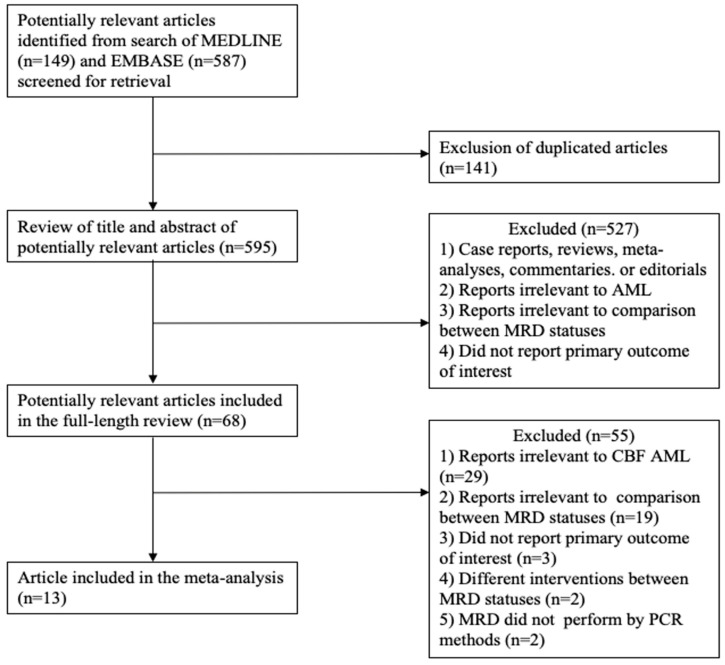
Flow-chart of literature review and selection process.

A total of 736 potentially relevant articles were identified during a search of the MEDLINE (*n* = 149) and EMBASE (*n* = 587) databases. Of those, 141 duplicate articles were excluded. The 595 remaining potentially relevant articles were then reviewed by two investigators, and case reports, reviews, meta-analyses, commentaries, and editorials were excluded. Remaining reports that were unrelated to AML, that lacked data from a comparison between MRD negative and positive status, or that did not report the primary outcome of interest were also excluded. A full-length review of the remaining 68 potentially relevant articles was then performed. This review excluded 55 additional reports that were unrelated to CBF-AML, that lacked a comparison between MRD statuses, that did not report the primary outcome of interest, that reported different interventions between the two MRD status groups, or that evaluated MRD status via a method other than polymerase chain reaction (PCR). The remaining 13 studies were included in this meta-analysis. The literature review and selection process are described in Figure 1.

### 3.1. Baseline Patient Characteristics

A total of 694 CBF-AML patients from the 13 included studies were enrolled. Of those, 260 cases were in the MRD positive group, and 434 cases were allocated to the MRD negative group. Among the entire cohort, 361 patients had the *RUNX1-RUNX1T1* fusion gene, 186 patients had the *CBFB-MYH11* fusion gene, and 147 patients had either *RUNX1-RUNX1T1* or *CBFB-MYH11.* While treatment regimens varied among studies, 7 days of cytarabine and 3 of anthracycline were commonly for induction therapy. The strategy of MRD monitoring varied among studies, with some starting after induction, some first during consolidation, and others even after consolidation. A final parameter which was highly variable between studies was the cutoff level for MRD positivity, which varied from 10^−2^ to 10^−6^ according to the RT-qPCR method used in each study. Baseline patient characteristics, CBF types, treatment protocol, time of MRD monitoring, MRD cutoff, source of MRD, and study period for all included articles are shown in Table 1.

### 3.2. Clinical Outcome

**Figure 2:**
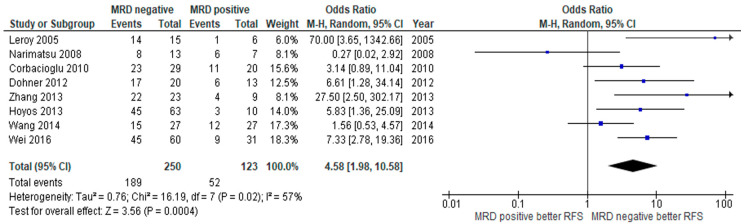
Forest plot of studies comparing relapse-free survival (RFS) between MRD negative patients and MRD positive patients.

**Figure 3:**
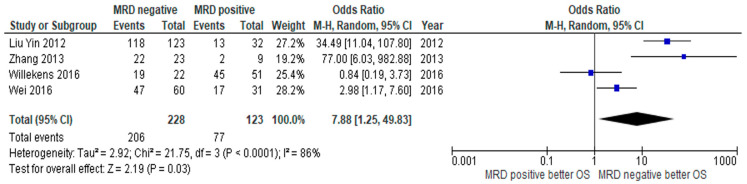
Forest plot of studies comparing overall survival (OS) between MRD negative patients and MRD positive patients.

**Figure 4:**
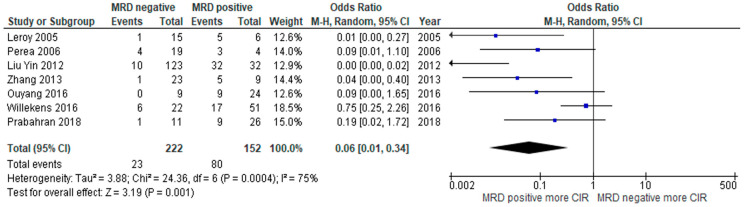
Forest plot of studies comparing cumulative incidence of relapse (CIR) between MRD negative patients and MRD positive patients.

The primary outcomes of interest in this study were RFS and/or OS. Eight of 13 studies reported RFS compared between MRD negative and positive status. The MRD negative group had superior RFS compared to those with MRD positivity, with a pooled odds ratio (OR) of 4.58 (95% confidence interval (CI): 1.98–10.58, *p* = 0.0004, I^2^ = 57%) (Figure 2) [16,18,19,20,22,23,24,25]. OS was reported in four studies with similar observed benefits among MRD negative patients, with a pooled OR of 7.88 (95% CI: 1.25–49.83, *p* = 0.03, I^2^ = 86%) (Figure 3) [21,23,24,27]. The CIR was statistically significantly lower in the MRD negative group than in the positive group, with a pooled OR of 0.06 (95% CI: 0.01–0.34, *p* = 0.001, I^2^ = 75%) (Figure 4) [16,17,21,24,26,27,28].

### 3.3. Subgroup Analysis

**Figure 5:**
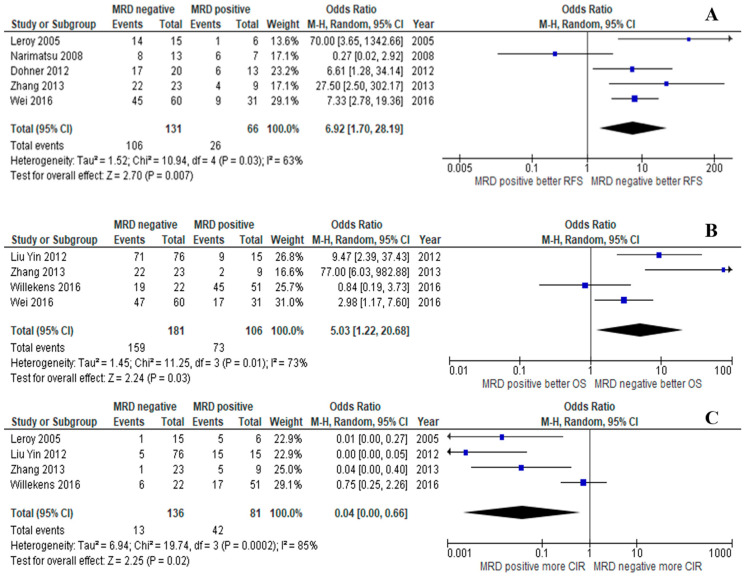
Forest plot of studies comparing (**A**) relapse-free survival, (**B**) overall survival, or (**C**) cumulative incidence of relapse between MRD negative patients and MRD positive patients among patients with the *RUNX1-RUNX1T1* fusion gene.

**Figure 6:**
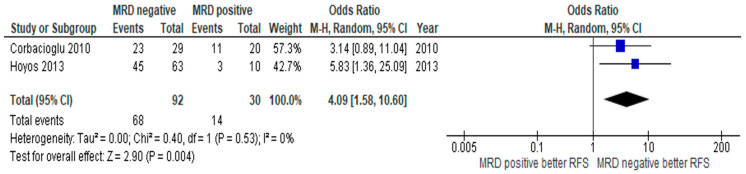
Forest plot of studies which compare relapse-free survival between MRD negative patients and MRD positive patients among patients with the *CBFB-MYH11* fusion gene.

In subgroup analysis of *RUNX1-RUNXT1* patients, the RFS of patients with MRD negativity was significantly better than in MRD positive patients, with a pooled OR of 6.92 (95% CI: 1.70–28.19, *p* = 0.007, I^2^ = 63%) (Figure 5A) [16,18,20,23,24]. Similarly, OS in patients with *RUNX1-RUNXT1* with negative MRD was higher than those with positive MRD, with a pooled OR of 5.03 (95% CI: 1.22–20.68, *p* = 0.03, I^2^ = 73%) (Figure 5B) [21,23,24,27]. The CIR in the MRD negative group was significantly lower in the MRD negative group, with a pooled OR of 0.04 (95% CI: 0.00–0.66, *p* = 0.02, I^2^ = 85%) (Figure 5C) [16,21,24,27]. A similar result was observed in subgroup analysis of *CBFB-MYH11*. Thus, RFS was significantly higher in the MRD negative group compared to the positive group, with a pooled OR of 4.09 (CI: 1.58–10.60, *p* = 0.004, I^2^ = 0%) (Figure 6) [19,22].

We next performed a subgroup analysis to identify the best timing for MRD assessment. For the MRD monitoring after induction subgroup, the MRD negative group had statistically significantly better RFS than the MRD positive group, with a pooled OR of 8.34 (95% CI: 3.86–18.02, *p* < 0.0001, I^2^ = 0%), and lower CIR in the MRD negative group was observed when compared with another group with a pooled OR of 0.09 (95% CI: 0.02–0.37, *p* = 0.0008, I^2^ = 0%) (Supplementary Data 3) [22,23,24,26,28]. For the MRD monitoring after consolidation therapy subgroup, there were no differences in OS and RFS between both groups; however, patients who achieved MRD negativity had lower CIR compared with those who remained MRD positive, with a pooled OR of 0.04 (95% CI: 0.00–0.91, *p* = 0.04, I^2^ = 87%) (Supplementary Data 4) [16,17,18,19,21,25,27].

We finally evaluated the cutoff as a parameter of MRD assessment. The most commonly used in this meta-analysis was 1 × 10^−3^ [17,18,25,26,28]; therefore, we also performed subgroup analysis, selecting only the studies with this cutoff. The MRD negative group had inferior CIR outcome when compared to the patients with MRD positivity, with a pooled OR of 0.12 (95% CI: 0.03–0.52, *p* = 0.004, I^2^ = 0%). However, there was no statistically significant difference in RFS outcome between both groups with this cutoff point (Supplementary Data 5) [17,18,25,26,28].

## 4. Discussion

During post-treatment follow-up of AML patients, persistent leukemic clones may lead to relapsed disease [29]. To recognize a lower concentration of leukemic cells, higher sensitivity investigations are required to monitor residual cancer in bone marrow, which is referred to as measurable residual disease (MRD) [30]. Two methods of evaluating MRD that are widely accepted in clinical trials and clinical practice are multiparametric flow cytometry (MFC) and RT-qPCR [31]. However, due to the immunophenotypic and molecular heterogeneity of AML clones, and the relative unavailability of these tests in many settings and countries, MRD testing is not routinely performed. Furthermore, no published guidelines recommend the optimal time for MRD investigation. MRD from RT-qPCR measures the amplification of a targeted DNA molecule [31]. This technique has enough sensitivity to detect one malignant cell in 10^4^ to 10^6^ leukocytes. It can also detect target genes and is considered the gold standard for MRD detection [5].

This is the first meta-analysis of qualitative MRD assessment in CBF-AML (*RUNX1-RUNX1T1* or *CBFB-MYH11)* and association with clinical outcomes. Our results show that MRD negative CBF-AML patients have significantly better OS/RFS. In addition, the CIR was found to be significantly lower in the MRD negative group than in the MRD positive group. Subgroup analysis of each fusion gene (*RUNX1-RUNX1T1* or *CBFB-MYH11*) found OS, RFS, and CIR to be significantly better in the MRD negative group.

According to European Society for Blood and Marrow Transplantation (EBMT) recommendation, MRD monitoring in AML is suggested after induction chemotherapy [32]. MRD status at post-remission treatment in first CR for favorable cytogenetic risk AML, including *RUNX1-RUNX1T1* and *CBFB-MYH11*, can be used to guide further management [33]. In MRD negative patients, post-remission treatment consists of consolidation chemotherapy or auto-HSCT, whereas allo-HSCT is preferred in those that are MRD positive [34]. Consistent with the findings of this meta-analysis, MRD positive patients have an inferior outcome. This analysis thus clearly emphasizes the importance of MRD monitoring by RT-qPCR technique in CBF-AML as a routine work-up for prognostication of these patients and for clinical decision-making.

### Limitations

This study has some limitations. First, the MRD cutoff levels vary among the included studies according to their experience and institutional policy. We were, therefore, unable to identify the optimal cutoff point for MRD assessment in CBF-AML. We were also unable to determine the best time point in the evolution of treatment to evaluate MRD. Nonetheless, a previous study proposes suitable timing for MRD assessment post-induction, post-consolidation, and then tri-monthly during the first 18 months of follow-up [21]. Eight of 13 studies evaluated MRD after the consolidation phase, and the remaining studies assessed MRD after induction. This highlights the need for a prospective study to identify the optimal time point for and the extent of MRD assessment in CBF-AML patients.

## 5. Conclusions

MRD negative CBF-AML patients had better OS, RFS, and CIR than their MRD positive CBF-AML counterparts. These results suggest that MRD assessment should be a routine investigation in clinical practice in this AML subset, even in strained economies, since it improves clinical decision-making and can result in patients avoiding expensive procedures like stem cell transplantation.

## Figures and Tables

**Table 1:** MRD status, CBF types, treatment protocol, HSCT, time of MRD monitoring, MRD cutoff, source of MRD, and the study period for all included articles.

References	Numbers		CBF Types	Treatment	HSCT	Time of MRD Monitoring	MRD Cutoff	Source of MRD	Study Period
	MRD Positive	MRD Negative							
Leroy 2005 [16]	6	15	*RUNX1-RUNX1T1* (*n* = 21)	Induction: daunorubicin, cytarabine and mitoxantrone Consolidation: mitoxantrone, cytarabine and idarubicin, cytarabine	Allo-HSCT	Pc	10^−5^	PB or BM	1994–2001
Perea 2006 [17]	4	19	*RUNX1-RUNX1T1* or *CBFB-MYH11* (*n* = 23)	Induction: idarubicin, etoposide, cytarabine Intensification: cytarabine and mitoxantrone Consolidation: HIDAC	Allo-HSCT (secondary AML)	Pc	10^−3^	BM	NA
Narimatsu 2008 [18]	7	13	*RUNX1-RUNX1T1* (*n* = 20)	Induction: idarubicin, cytarabine or daunorubicin, cytarabine Consolidation: HIDAC, IDAC	NR	Pc (cycle1)	10^−3^	BM	2000–2005
Corbacloglu 2010 [19]	20	29	*CBFB-MYH11* (*n* = 49)	Induction: ICEx2, ICE then S-HAM or HAM Consolidation: HIDAC	Auto-HSCT Allo-HSCT	Pc (cycle3)	10^−5^	BM	1992–2006
Dohner 2012 [20]	13	20	*RUNX1-RUNX1T1* (*n* = 33)	Induction: ICEx2 Consolidation: HIDAC	Auto-HSCT Allo-HSCT	Pc1-Pc	10^−6^	BM	1992–2004
Liu Yin 2012 [21] (1)	15	76	*RUNX1-RUNX1T1* (*n* = 91)	Induction: daunorubicin, cytarabine and/or etoposide or FLAG-Idarubicin and/or GO Consolidation: MACE or MIDAC or IDAC/HIDAC and/or GO	NR	Pc (cycle4)	5 × 10^−3^	BM	2002–2009
Liu Yin 2012 [21] (2)	17	47	*CBFB-MYH11* (*n* = 64)	Induction: daunorubicin, cytarabine and/or etoposide or FLAG-Idarubicin and/or GO Consolidation: MACE or MIDAC or IDAC/HIDAC and/or GO	NR	Pc (cycle4)	5 × 10^−4^	BM	2002–2009
Hoyos 2013 [22]	10	63	*CBFB-MYH11* (*n* = 73)	Induction: idarubicin, cytarabine and etoposide Consolidation: mitoxantone and cytarabine, HIDAC	Auto-HSCT	Pi	10^−2^	BM	1999–2012
Wei 2016 [23]	31	60	*RUNX1-RUNX1T1* (*n* = 91)	Induction: homoharringtonine, cytarabine, daunorubicin Consolidation: HIDAC, IDAC	Allo-HSCT	Pi	10^−2^	NA	2010–2016
Zhang 2013 [24]	9	23	*RUNX1-RUNX1T1* (*n* = 32)	Induction: cytarabine based chemotherapy Consolidation: HIDAC, IDAC	HSCT	Pi	10^−4^	BM	2004–2011
Wang 2014 [25]	27	27	*RUNX1-RUNX1T1* or *CBFB-MYH11* (*n* = 54)	Induction: cytarabine, daunorubicin/idarubicin Consolidation: IDAC	No	Pc (cycle4)	10^−3^	BM	NA–2013
Ouyang 2016 [26]	24	9	*RUNX1-RUNX1T1* or *CBFB-MYH11* (*n* = 33)	Induction: FLAG-idarubicin Consolidation: FLAG or decitabine	HSCT (Relapse)	Pi	10^−3^	BM	2012–2014
Willekens 2016 [27]	51	22	*RUNX1-RUNX1T1* (*n* = 73)	Induction: cytarabine, daunorubicin Consolidation: HIDAC	No	Pc (cycle3)	10^−5^	BM	2007–2010
Prabahran 2018 [28]	26	11	*RUNX1-RUNX1T1* or *CBFB-MYH11* (*n* = 37)	Induction: cytarabine, idarubicin/daunorubicin, HIDAC, ICE, FLAG, IDAC, MIDAC Consolidation: HIDAC, ICE, IDAC, FLAG, MIDAC	No	Pi	10^−3^	BM	2001–2012

**Abbreviations:***Allo-* allogeneic, *Auto-* autologous, *BM* bone marrow, *CBF* core-binding factor, *FLAG* filgrastim fludarabine cytarabine, *GO* gemtuzumab ozogamicin, *HAM* high-dose cytarabine mitoxantone, *HIDAC* high-dose cytarabine, *HSCT* hematopoietic stem cell transplantation, *ICE* idarubicin cytarabine etoposide, *IDAC* intermediate-dose cytarabine, *MACE* amsacrine cytarabine etoposide, *MIDAC* mitoxantone cytarabine, *MRD* measurable residual disease, *NR* not reported, *NA* not applicable, *Pi* post induction, *PB* peripheral blood, *Pc* post consolidations, *Pc1* post consolidation cycle 1, *Pc1-Pc* during consolidation, *S-HAM* sequential high-dose cytarabine and mitoxantone.

## Supplementary Materials

The following are available online at https://www.mdpi.com/2075-4426/10/4/250/s1, Data 1: search strategy, Data 2: PRISMA 2009 Checklist, Data 3: Forest plots of studies that compared: (A) relapse-free survival; (B) overall survival; (C) cumulative incidence of relapse among patients who underwent MRD negative versus MRD positive in patients after induction therapy, Data 4: Forest plots of studies that compared: (A) relapse-free survival; (B) overall survival; (C) cumulative incidence of relapse among patients who underwent MRD negative versus MRD positive in patients after consolidation therapy, Data 5: Forest plots of studies that compared: (A) overall survival; (B) cumulative incidence of relapse among patients who underwent MRD negative versus MRD positive in patients with the cutoff MRD of 1 × 10^−3^.
