# Supplementary material for: Clinical Outcomes Based on Measurable Residual Disease Status in Patients with Core-Binding Factor Acute Myeloid Leukemia: A Systematic Review and Meta-Analysis
Source: J Pers Med. 2020 Nov 26;10(4):250. doi: 10.3390/jpm10040250 (PMC7711894; doi:10.3390/jpm10040250)
Supplement: Supplementary file 1 [file jpm-10-00250-s001.pdf]

Supplementary data 1

Search strategy

EMBASE

1. 'acute myeloid leukemia'/exp OR 'acute myeloid leukemia'
2. 'acute myeloid leukaemia'/exp OR 'acute myeloid leukaemia'
3. 'minimal residual disease'/exp OR 'minimal residual disease'
4. 'minimal measurable disease'/exp OR 'minimal measurable disease'
5. #1 OR #2
6. #3 OR #4
7. #5 AND #6

Medline

1. acute myeloid leukemia.mp. or exp Leukemia, Myeloid, Acute/
2. acute myeloid leukaemia.mp. or exp Leukaemia, Myeloid, Acute/
3. minimal residual disease.mp. or exp Disease, Residual, Minimal/
4. minimal measurable disease.mp. or exp Disease, Measurable, Minimal/
5. 1 or 2
6. 3 or 4
7. 5 and 6

Supplementary data 2 PRISMA checklist

PRISMA 2009 Checklist

Section/topic	#	Checklist item	Reported on page #
**TITLE**			
Title	1	Identify the report as a systematic review, meta-analysis, or both.	1
**ABSTRACT**			
Structured summary	2	Provide a structured summary including, as applicable: background; objectives; data sources; study eligibility criteria, participants, and interventions; study appraisal and synthesis methods; results; limitations; conclusions and implications of key findings; systematic review registration number.	1
**INTRODUCTION**			
Rationale	3	Describe the rationale for the review in the context of what is already known.	1
Objectives	4	Provide an explicit statement of questions being addressed with reference to participants, interventions, comparisons, outcomes, and study design (PICOS).	2
**METHODS**			
Protocol and registration	5	Indicate if a review protocol exists, if and where it can be accessed (e.g., Web address), and, if available, provide registration information including registration number.	2 and supplementary data 1
Eligibility criteria	6	Specify study characteristics (e.g., PICOS, length of follow-up) and report characteristics (e.g., years considered, language, publication status) used as criteria for eligibility, giving rationale.	2
Information sources	7	Describe all information sources (e.g., databases with dates of coverage, contact with study authors to identify additional studies) in the search and date last searched.	2
Search	8	Present full electronic search strategy for at least one database, including any limits used, such that it could be repeated.	2 and supplementary data 1
Study selection	9	State the process for selecting studies (i.e., screening, eligibility, included in systematic review, and, if applicable, included in the meta-analysis).	2
Data collection process	10	Describe method of data extraction from reports (e.g., piloted forms, independently, in duplicate) and any processes for obtaining and confirming data from investigators.	2
Data items	11	List and define all variables for which data were sought (e.g., PICOS, funding sources) and any assumptions and simplifications made.	2
Risk of bias in individual studies	12	Describe methods used for assessing risk of bias of individual studies (including specification of whether this was done at the study or outcome level), and how this information is to be used in any data synthesis.	3
Summary measures	13	State the principal summary measures (e.g., risk ratio, difference in means).	3
Synthesis of results	14	Describe the methods of handling data and combining results of studies, if done, including measures of consistency (e.g., I^2) for each meta-analysis.	3

Section/topic	#	Checklist item	Reported on page #
Risk of bias across studies	15	Specify any assessment of risk of bias that may affect the cumulative evidence (e.g., publication bias, selective reporting within studies).	N/A due to limited included studies
Additional analyses	16	Describe methods of additional analyses (e.g., sensitivity or subgroup analyses, meta-regression), if done, indicating which were pre-specified.	N/A
**RESULTS**			
Study selection	17	Give numbers of studies screened, assessed for eligibility, and included in the review, with reasons for exclusions at each stage, ideally with a flow diagram.	3
Study characteristics	18	For each study, present characteristics for which data were extracted (e.g., study size, PICOS, follow-up period) and provide the citations.	3
Risk of bias within studies	19	Present data on risk of bias of each study and, if available, any outcome level assessment (see item 12).	Table 1
Results of individual studies	20	For all outcomes considered (benefits or harms), present, for each study: (a) simple summary data for each intervention group (b) effect estimates and confidence intervals, ideally with a forest plot.	Table 1
Synthesis of results	21	Present results of each meta-analysis done, including confidence intervals and measures of consistency.	3-4
Risk of bias across studies	22	Present results of any assessment of risk of bias across studies (see Item 15).	N/A due to limited included studies
Additional analysis	23	Give results of additional analyses, if done (e.g., sensitivity or subgroup analyses, meta-regression [see Item 16]).	N/A
**DISCUSSION**			
Summary of evidence	24	Summarize the main findings including the strength of evidence for each main outcome; consider their relevance to key groups (e.g., healthcare providers, users, and policy makers).	4-5
Limitations	25	Discuss limitations at study and outcome level (e.g., risk of bias), and at review-level (e.g., incomplete retrieval of identified research, reporting bias).	5
Conclusions	26	Provide a general interpretation of the results in the context of other evidence, and implications for future research.	5
**FUNDING**			
Funding	27	Describe sources of funding for the systematic review and other support (e.g., supply of data); role of funders for the systematic review.	6

From: Moher D, Liberati A, Tetzlaff J, Altman DG, The PRISMA Group (2009). Preferred Reporting Items for Systematic Reviews and Meta-Analyses: The PRISMA Statement. PLoS Med 6(7): e1000097. doi:10.1371/journal.pmed1000097

For more information, visit: www.prisma-statement.org.

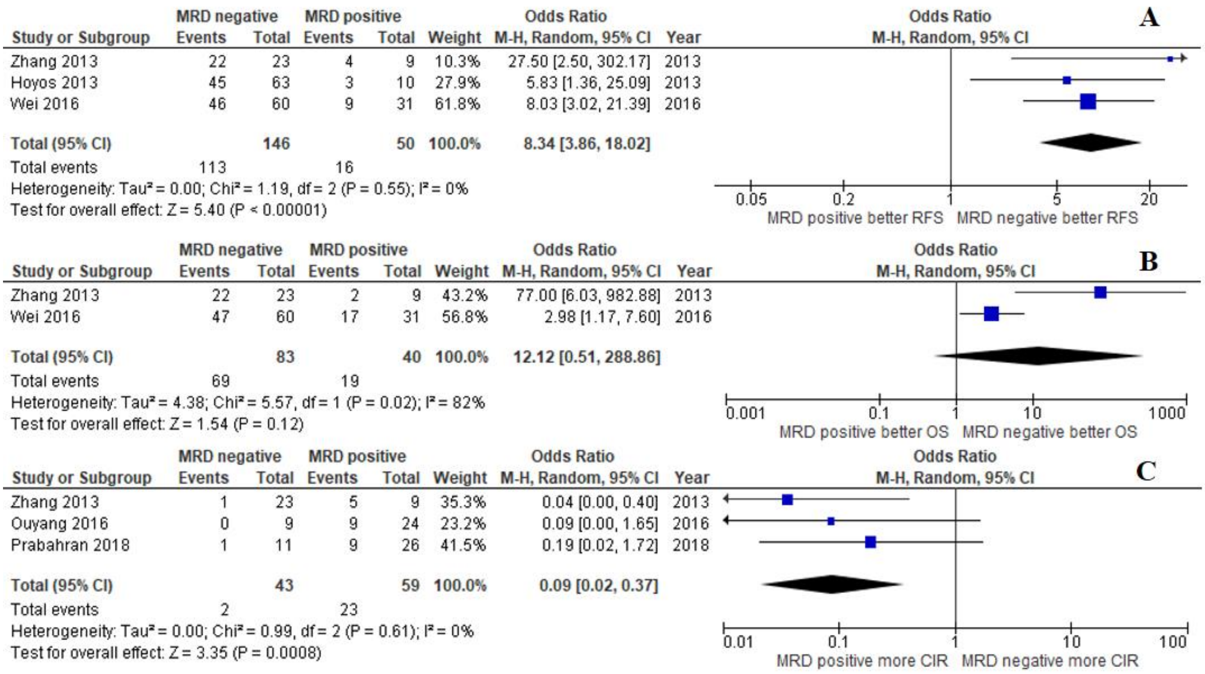

Supplementary data 3 Forest plots of studies that compared: A) relapse-free survival; B) overall survival; C) cumulative incidence of relapse among patients who underwent MRD negative versus MRD positive in patients after induction therapy

Study or Subgroup	MRD negative Events	MRD negative Total	MRD positive Events	MRD positive Total	Weight	Odds Ratio M-H, Random, 95% CI	Year
Leroy 2005	14	15	1	6	15.4%	70.00 [3.65, 1342.66]	2005
Narimatsu 2008	8	13	6	7	19.6%	0.27 [0.02, 2.92]	2008
Corbacioglu 2010	23	29	11	20	31.4%	3.14 [0.89, 11.04]	2010
Wang 2014	15	27	12	27	33.5%	1.56 [0.53, 4.57]	2014
**Total (95% CI)**		**84**		**60**	**100.0%**	**2.47 [0.57, 10.77]**	
Total events	60		30				

Heterogeneity: Tau² = 1.38; Chi² = 8.95, df = 3 (P = 0.03); I² = 66%
Test for overall effect: Z = 1.21 (P = 0.23)

Study or Subgroup	MRD negative Events	MRD negative Total	MRD positive Events	MRD positive Total	Weight	Odds Ratio M-H, Random, 95% CI	Year
Liu Yin 2012	118	123	13	32	50.8%	34.49 [11.04, 107.80]	2012
Willekens 2016	19	22	44	51	49.2%	1.01 [0.24, 4.32]	2016
**Total (95% CI)**		**145**		**83**	**100.0%**	**6.07 [0.19, 198.63]**	
Total events	137		57				

Heterogeneity: Tau² = 5.89; Chi² = 14.24, df = 1 (P = 0.0002); I² = 93%
Test for overall effect: Z = 1.01 (P = 0.31)

Study or Subgroup	MRD negative Events	MRD negative Total	MRD positive Events	MRD positive Total	Weight	Odds Ratio M-H, Random, 95% CI	Year
Leroy 2005	1	15	5	6	23.3%	0.01 [0.00, 0.27]	2005
Perea 2006	4	19	3	4	24.7%	0.09 [0.01, 1.10]	2006
Liu Yin 2012	10	123	32	32	23.6%	0.00 [0.00, 0.02]	2012
Willekens 2016	6	22	17	51	28.4%	0.75 [0.25, 2.26]	2016
**Total (95% CI)**		**179**		**93**	**100.0%**	**0.04 [0.00, 0.91]**	
Total events	21		57				

Heterogeneity: Tau² = 8.60; Chi² = 23.39, df = 3 (P < 0.0001); I² = 87%
Test for overall effect: Z = 2.02 (P = 0.04)

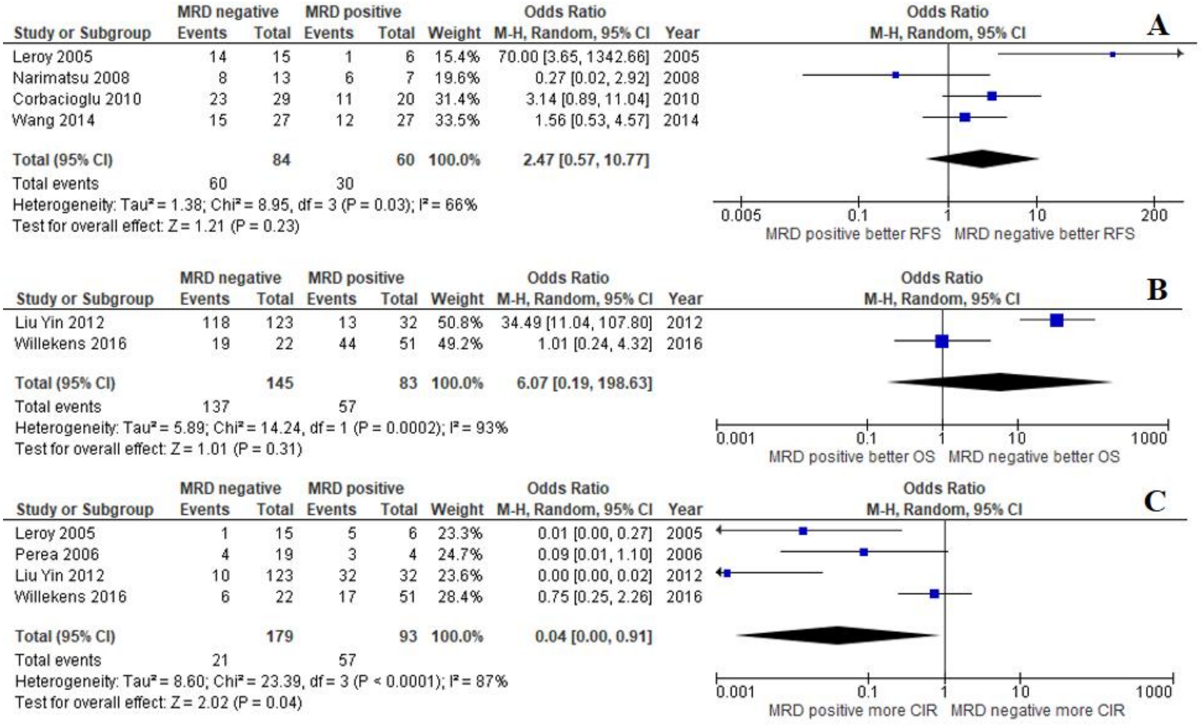

Supplementary data 4 Forest plots of studies that compared: A) relapse-free survival; B) overall survival; C) cumulative incidence of relapse among patients who underwent MRD negative versus MRD positive in patients after consolidation therapy

A

Study or Subgroup	MRD negative Events	MRD negative Total	MRD positive Events	MRD positive Total	Weight	Odds Ratio M-H, Random, 95% CI	Year
Narimatsu 2008	8	13	6	7	31.1%	0.27 [0.02, 2.92]	2008
Wang 2014	15	27	12	27	68.9%	1.56 [0.53, 4.57]	2014
**Total (95% CI)**		**40**		**34**	**100.0%**	**0.90 [0.18, 4.51]**	
Total events	23		18				

Heterogeneity: Tau² = 0.68; Chi² = 1.76, df = 1 (P = 0.18); I² = 43%
Test for overall effect: Z = 0.13 (P = 0.90)

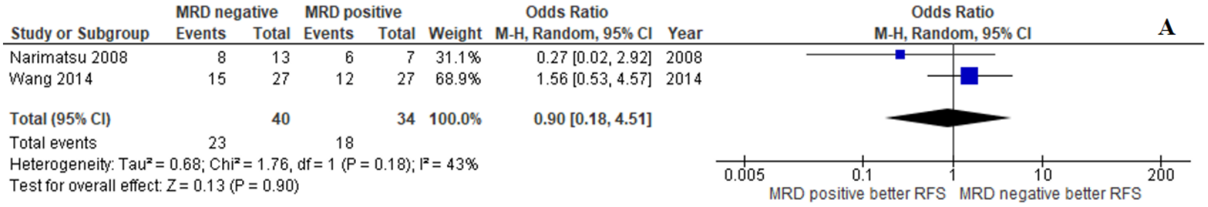

B

Study or Subgroup	MRD negative Events	MRD negative Total	MRD positive Events	MRD positive Total	Weight	Odds Ratio M-H, Random, 95% CI	Year
Perea 2006	4	19	3	4	33.1%	0.09 [0.01, 1.10]	2006
Ouyang 2016	0	9	9	24	24.0%	0.09 [0.00, 1.65]	2016
Prabahran 2018	1	11	9	26	43.0%	0.19 [0.02, 1.72]	2018
**Total (95% CI)**		**39**		**54**	**100.0%**	**0.12 [0.03, 0.52]**	
Total events	5		21				

Heterogeneity: Tau² = 0.00; Chi² = 0.27, df = 2 (P = 0.88); I² = 0%
Test for overall effect: Z = 2.85 (P = 0.004)

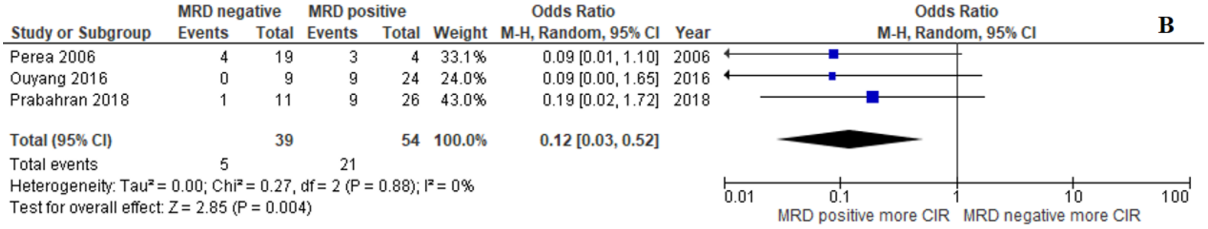

Supplementary data 5 Forest plots of studies that compared: A) overall survival; B) cumulative incidence of relapse among patients who underwent MRD negative versus MRD positive in patients with the cut-off MRD of $1x10^{-3}$
